# Common clinical findings identified in working equids in low- and middle-income countries from 2005 to 2021

**DOI:** 10.1371/journal.pone.0304755

**Published:** 2024-06-05

**Authors:** Mathilde S. Merridale-Punter, Anke K. Wiethoelter, Charles M. El-Hage, Cameron Patrick, Peta L. Hitchens

**Affiliations:** 1 Melbourne Veterinary School, Faculty of Science, University of Melbourne, Parkville, Victoria, Australia; 2 Statistical Consulting Centre, School of Mathematics and Statistics, University of Melbourne, Parkville, Victoria, Australia; 3 Equine Lameness and Imaging Centre, Melbourne Veterinary School, University of Melbourne, Werribee, Victoria, Australia; Benha University Faculty of Veterinary Medicine, EGYPT

## Abstract

Despite several millions of working equids worldwide, there are few published studies regarding the epidemiology of their health and welfare. Data collected by non-governmental organisations (NGOs) operating in the working equid sphere therefore have important epidemiological value and could be used towards animal health surveillance. The aim of this study was to identify common clinical findings and mortality patterns of working equids in low- and middle-income countries and investigate their epidemiology using data collected from an international NGO. A retrospective analysis was conducted to determine the proportion of clinical findings and mortality risk by equid species, year and region. Negative binomial regression models were generated to investigate differences in mortality risk and proportion of key clinical findings between equid species, hemispheres and calendar month. A total of 4,313,606 presentations were reported from 14 countries between January 2005 and March 2021 (mean 22,121; SD ± 7,858 per month). Wounds and abscesses were the most reported clinical finding for all equid species (mean proportion 35%; SD ±0.19 of all findings). A higher proportion of wounds (mean proportion 41.7%; SD±0.2) was recorded in donkeys than mules or horses (P<0.001). Mules had higher reported mortality risk (1.2%; 95% CI 0.94–1.46%) than horses (0.4%; 95% CI 0.36–0.55%; p<0.001) or donkeys (0.2%; 95% CI 0.14–0.22%). Work-related wounds were the predominant finding in working equids, particularly so in donkeys. Prevention strategies should focus on improvements to work equipment and practices for all equids. Future investigations required include refinement of diagnostic approaches for donkeys and investigation of risk factors to understand the higher mortality in mules. Routine monitoring of clinical findings reported by national or international NGOs could be included in animal health surveillance strategies, although standardisation of data for this purpose is needed so that changes in prevalence following implementation of prevention strategies can be monitored.

## Introduction

Low- and middle-income countries (LMICs) contain some of the largest equid populations globally [1–3]. Working equids are widely used in many LMICs, providing an important source of income and domestic support to households [4–7]. However, socioeconomic pressures of LMICs frequently result in working equids being overworked often to the extremes of their physiological limits [8, 9]. As a result of these and other pressures, health problems are highly prevalent in this group of animals [4, 10–12]. Because of the role they play in their communities, such as supporting agriculture, food and water security and income-generating activities [4, 6, 13], the implications of compromised working equid health are a real One Health issue, which go beyond animal welfare and extend to the wellbeing of the communities that rely on them [6, 14]. Compounding this equid welfare issue is the reality that, in many LMICs, actual or relative access of working equids to veterinary services is often limited, and the existing veterinary infrastructure tends to be targeted at livestock species [4]. Additionally, a lack of financial incentives to practitioners for treating equids and the limited availability of many equid medicines in several LMICs also hinders the training of veterinary professionals in equid disciplines [4]. This further aggravates the problem and widens the gap between equids and other species in terms of disease prevention, treatment and surveillance. International non-governmental organisations (NGOs) aim to bridge this gap by providing direct or indirect support to local veterinary services, facilitating capacity building to veterinary professionals or creating infrastructure to support the health and welfare of working equids [15–18].

NGOs supporting working equids collect different forms of animal health data as part of their operations and monitoring activities [15–18]. They often have access to various populations of working equids that are underrepresented in global animal health epidemiological data. As such, routine monitoring of veterinary data from NGOs supporting working equids internationally may provide valuable insight into health issues faced by working equids, which are otherwise not available in most national disease records.

The aim of this study was therefore to identify common clinical findings of working equids of LMICs using existing health data from an international NGO supporting working equids. The specific objectives were to (1) describe the predominant clinical findings of equids presenting to a veterinary service provider in LMICs; (2) investigate how the occurrence of clinical findings varies according to country, equid species, year and other factors of interest; and (3) investigate patterns of mortality in working equids.

An improved understanding of issues affecting working equid health and welfare would enable development of evidence-based recommendations for governments and NGOs conducting national or international prevention programmes, informing strategy and preparedness. In addition, routine monitoring of such data could not only enable operational monitoring but also contribute more effectively to disease surveillance in working equids of LMICs. As such, we have included recommendations to enable this data to be used for animal health surveillance purposes.

## Materials and methods

Approval from the Animal Ethics Committee of the University of Melbourne for activities involving retrospective analysis of clinical veterinary data is not required *(Ethics Committee consulted on the 22nd March 2021)*.

### Data sources

This is a retrospective cross-sectional study, using existing anonymous and non-identifiable data from an international NGO, collected as part of their operational monitoring and made available for this research through a data sharing agreement. Data included in this study comprise clinical presentations and findings of animals brought to veterinary clinics supported by the NGO and situated in 14 LMICs in Africa and South Asia. Data was collected by the NGO’s veterinary staff or partners on the ground, for all working animals attended to at fixed or mobile clinics and entered into a standardised reporting template submitted to their headquarters monthly. To standardize data collected by different teams and increase reporting consistency, the same reporting template was used by all countries with the same project type and the NGO provided guidelines on how to complete the template.

Monthly veterinary reports from all projects providing direct veterinary care to working animals active between January 2005 and March 2021 that were available in the NGO’s archives were retrieved and compiled. From these, only data referring to equid species (horses, donkeys and mules) from projects reporting on a monthly basis on standardised templates were included in analysis. Other narrative reports from the NGO were consulted as needed to provide additional context and clarification.

The monthly veterinary reports contained two main segments, each with various sub-categories: (1) number of animals examined at the clinic and type of service delivered (treatment, treatment advice or euthanasia); and (2) number and type of clinical finding or treatment provided at the point of veterinary examination (for example “cough”, “pyrexia” or “tetanus vaccination”, where multiple clinical findings and treatments are possible for each individual animal). The following data fields were extracted and compiled: status (partnership or core project according to the contract terms and type of support provided by the NGO), country, clinic (region within the country), date, year, calendar month, section (type of intervention performed: clinical cases, preventive measures or distribution of equipment such as atraumatic harnessing and fly fringes), category of presentations or findings, specific clinical findings, number of clinical findings per equid species and number of animals attended to per equid species.

Reporting templates changed on three occasions over the 16-year study period (2016, 2018 and 2019), with changes to the nomenclature and categorisation of clinical findings. For the purpose of this study, to standardize categories and enable comparisons between the different reporting templates, clinical findings were re-classified into new standardized categories by co-author agreement (S1 File). In this study, the term ‘Findings Category’ was defined as the level of categorisation that includes multiple individual clinical findings and diagnosis bound by similar criteria: two distinct findings categories were created clustering specific clinical findings by body system affected (‘Findings Category 1’) and by alternative classifications such as infectious or parasitic findings (‘Findings Category 2’) to enable different perspectives and epidemiological analyses (Table 1 and S1 File). Findings Category 2 was only considered between May 2016 and March 2021 due to reporting changes occurring in this period.

**Table 1:** Mean proportion and standard deviation of clinical findings by category in relation to all clinical findings. Proportion of findings calculated for all countries included in a retrospective data analysis of clinical findings of working equids in low- and middle-income countries presenting to an international NGO between January 2005 and March 2021. The two finding categorisations used are described. Findings category 2 was analysed from May 2016 to March 2021 only.

Findings Category 1	Proportion	SD±	Findings Category 2 *May 2016 to Mar 2021 only	Proportion	SD±
Wounds and Abscesses	35%	0.19	Work-related Wounds	17.8%	0.16
Other Medical Cases	16.6%	0.20	Parasitism	14.6%	0.19
Musculoskeletal System	11.9%	0.09	Musculoskeletal System	13.3%	0.11
Respiratory System	10%	0.09	Other Wounds and Abscesses	12.5%	0.14
Skin Complaints	9.5%	0.13	Respiratory System	8.9%	0.09
Digestive System	6.5%	0.07	Ocular Cases	6.2%	0.07
Ocular and Aural Cases	5.7%	0.06	Other Medical Cases	5.9%	0.10
Oral Problems	3.3%	0.07	Oral Problems	5.9%	0.10
Surgical Cases	1.5%	0.07	Digestive System	5.8%	0.07
Cardiovascular System	1%	0.03	Non-parasitic Infectious Diseases	5%	0.11
Road Traffic Accidents	0.6%	0.02	Skin Complaints	1.8%	0.05
			Surgical Cases	1.5%	0.07
			Cardiovascular System	0.8%	0.04
			Road Traffic Accidents	0.6%	0.02
			Aural Problems	0.2%	0.01

Mortality was defined as any record of euthanasia or natural death, as differentiation between the two was not consistent during the study period. The total number of animals examined per species was defined as the number of ‘animals examined and treated’ plus the ‘animals examined with advice only’. Between 2005 and May 2016 only the number of clinical findings, animals examined with advice only and mortality were recorded, with no data collected for the numbers of animals examined and treated. Denominator data on the number of animals examined (‘examined and treated’ and ‘examined with advice only’) was available only from May 2016 to March 2021, thus a subset analysis was conducted for this period.

### Statistical analysis

Descriptive statistics were performed using R software version R4.2.1 (2021 R Core Team [19]). Frequency of distribution of animals examined and clinical findings by category were calculated. The mean proportion of clinical findings by category (numerator) in relation to the overall number of clinical findings over the same time period and geographical unit (denominator) was calculated and presented with the corresponding standard deviation (±SD). Changes in the proportion of finding categories over time were only analysed for findings category 1 and for categories consistent between the period from January 2005 to March 2021. Mortality risk was calculated as the number of mortalities recorded (numerator) over the number of animals examined (denominator). Mortality risk and proportion of clinical findings by category are reported by year, calendar month, country, hemisphere and species. A binary ‘epoch’ variable was created to reflect a major change in reporting template taking place from May 2016 and to investigate potential relationships with reporting template changes. Multivariable negative binomial regression models were used to investigate differences in the proportion of clinical findings and in mortality risk as model outcomes. Four models were created for each of the outcomes, adjusted for and investigating interaction terms between the predictors species, hemisphere, calendar month and date respectively. Date was modelled as a continuous variable to allow a linear effect of time to be assessed. The models incorporated AR(1) autocorrelation of error terms within each species, calendar month and hemisphere since counts in successive dates were correlated. Models investigating differences in the proportion of clinical findings included a log-transformed offset term treating all findings as the denominator and the findings of interest as the numerator, and were run both with and without the inclusion of epoch as a further interaction term to account for potential differences due to change of reporting template. Results reported refer to epoch adjusted models unless otherwise stated. Pairwise comparisons of the mean proportion of wounds and mortality risk for each species, calendar month and hemisphere were then performed; risk ratios (RR), mortality risk ratios (MRR) and their 95% confidence intervals (95% CI) are presented. Changes in the type of intervention performed over time were also investigated through a multivariable negative binomial regression model including section (preventive measures, clinical cases or distribution of equipment), date and epoch as predictors and using an AR(1) autocorrelation of error terms within each section. The R package glmmTMB (version 1.4.1) was used to fit these models [20]. Statistical significance was considered at p<0.05.

### Inclusivity in global research

Additional information regarding the ethical, cultural, and scientific considerations specific to inclusivity in global research is included in the Supporting Information (S2 File).

## Results

Equid health data collated between January 2005 and March 2021, in 66 locations from a total of 14 countries (Algeria, Botswana, Ethiopia, India, Jordan, Kenya, Mali, Mauritania, Morocco, South Africa, Syria, Tanzania, Tunisia and Zimbabwe) were analysed. Data for Ethiopia, Mali, Mauritania, Morocco and Tunisia was available for the complete 195-month period, while Jordan had data for 194 months, Syria for 98 months, Zimbabwe for 87 months and Botswana for 70 months. South Africa, Algeria, India, Tanzania and Kenya contributed data for 50, 47, 17, 14 and 12 months respectively.

**Fig 1:**
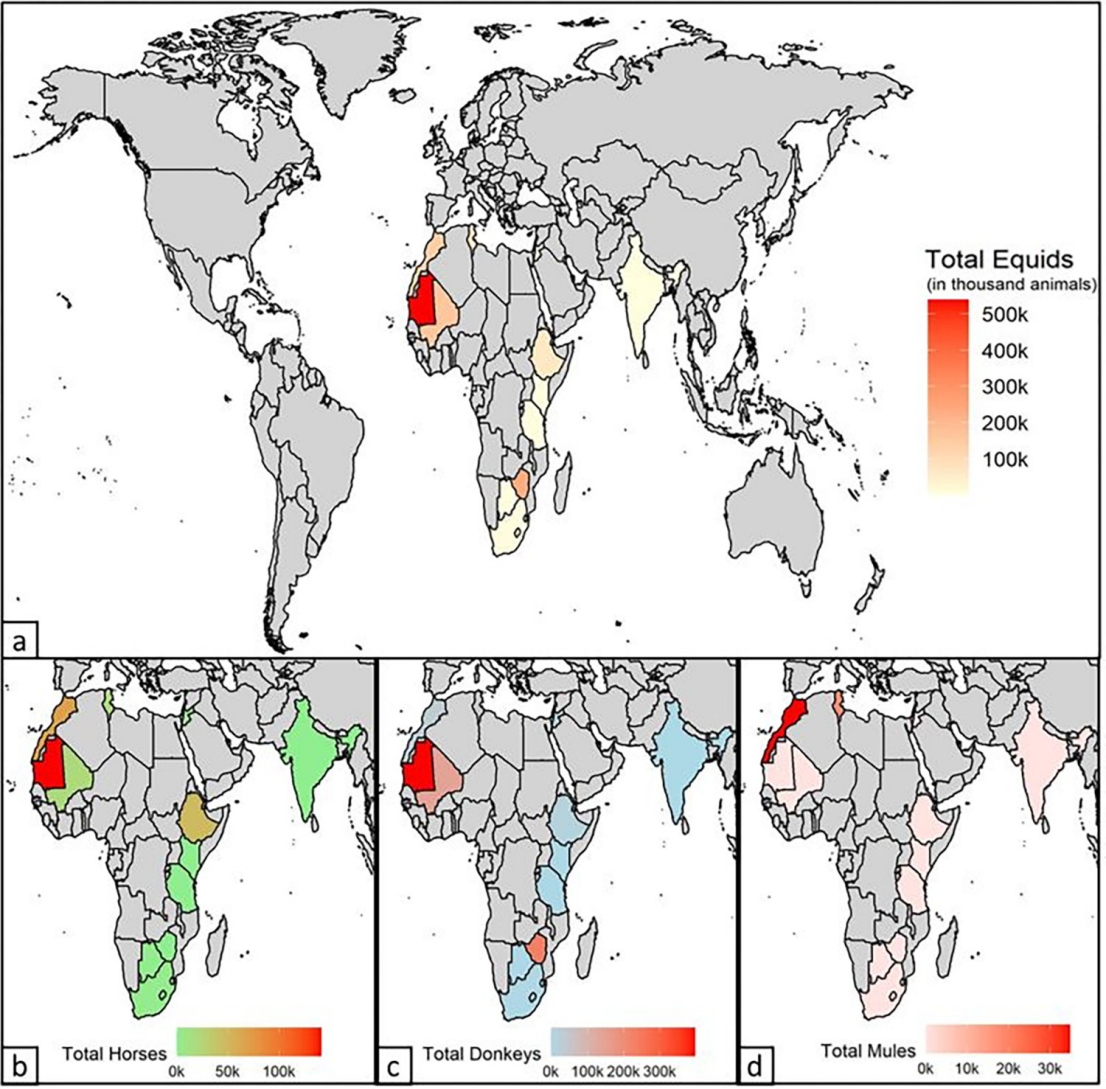
Overall number of equids examined per country (a) and number of horses (b), donkeys (c) and mules (d) examined per country in a retrospective data analysis of clinical findings of working equids in low- and middle-income countries presenting to an international NGO between May 2016 and March 2021. Created using R 4.2.1.

A total of 4,313,606 clinical findings, treatments, and equipment distributions were reported during the study period, with a mean of 22,121 (SD ± 7,858) per month between 2005 and 2021. A total of 1,226,412 equids presented during May 2016 to March 2021, of which 71.2% (872,905/1,226,412) were donkeys, 24.5% horses (300,680/1,226,412) and 4.3% (52,827/1,226,412) mules (S3 File). The number of animals examined between May 2016 and March 2021 ranged from 109 equids in Kenya to 529,826 equids in Mauritania. Donkeys were the predominant species presenting in Mauritania, Mali, South Africa, Tanzania, Tunisia and Zimbabwe, whereas horses were most commonly treated in Botswana, Ethiopia, India and Morocco. Mules were the least presented species of equids in all countries with the exception of Morocco where mules presented in higher proportion than donkeys (Fig 1 and S3 File).

The main type of veterinary intervention provided to working equids presenting to clinics during the study period were preventive measures (54.6%; 2,353,259/4,313,606), followed by treatment or treatment advice for clinical cases (41.6%; 1,795,877/4,313,606) and distribution of equipment (3.8%; 164,470/4,313,606). Algeria, India, Jordan, Morocco, Syria and Tunisia saw a higher proportion of clinical cases than preventive measures or distribution of equipment, while preventive measures were the predominant type of intervention in Botswana, Ethiopia, Kenya, Mali, Mauritania, South Africa, Tanzania and Zimbabwe (S3 File). Morocco had the highest monthly average of clinical cases (mean 3,076 ± 2,178) and distribution of equipment (mean 417 ± 291), while Mauritania saw the highest monthly average of preventive measures (mean 4,093 ± 1,620). The lowest monthly average of clinical cases and preventive measures was recorded in Kenya (mean 0.5 ±1; and 6 ± 10 respectively), and both India and Kenya recorded no distribution of equipment during the study period (S3 File). Overall, there was a decrease in equipment distribution over time (coef. -0. 84 per 100 months on natural log scale; 95% CI -1.34 to -0.34; p = 0.001).

When considering clinical cases only, a total of 1,795,877 clinical findings were recorded during the study period, ranging from six findings recorded for Kenya to 599,775 in Morocco. Botswana, Ethiopia, Jordan, India and Syria recorded more clinical findings in horses than other species, whereas all other countries recorded more clinical findings in donkeys. Mules were the species with the lowest number of clinical findings recorded in all countries, with the exception of Morocco where mules demonstrated the most clinical findings until 2016 (S3 File).

### Mortality

The overall equid mortality risk between May 2016 and March 2021 was 2.8 deaths per 1,000 animals (3,386/1,226,412), ranging from 1.9 deaths per 1,000 animals (664/354,714) in 2019 to 4 deaths per 1,000 animals (656/162,670) in 2017. Botswana reported the highest overall mortality risk (37 deaths per 1,000 animals; 102/2,744), followed by Morocco (15 deaths per 1,000 animals; 2,043/137,829), Ethiopia (7 deaths per 1,000 animals; 405/60,962), South Africa (5 deaths per 1000 animals; 28/6,229), Jordan (3 deaths per 1,000 animals; 25/7,360), Zimbabwe (1 death per 1,000 animals; 247/231,085), Mali (1 death per 1000 animals; 126/159,468), Mauritania (1 death per 1,000 animals; 356/529,826) and Tunisia (1 death per 1,000 animals; 54/89,067). India, Kenya and Tanzania recorded no mortalities. The mean mortality risk (MMR) per calendar month ranged from 5 deaths per 1,000 animals (±5.2) in December to 9 deaths (±8.9) in August (S3 File). Mean mortality risk was greater in the Southern Hemisphere (MMR = 18 deaths per 1,000 animals; 95% CI 13–26) compared to the Northern Hemisphere (MMR = 7 deaths per 1000 animals; 95% CI 6–8; p<0.001). Mean mortality risk did not change significantly over time and no differences were found per calendar month.

**Fig 2:**
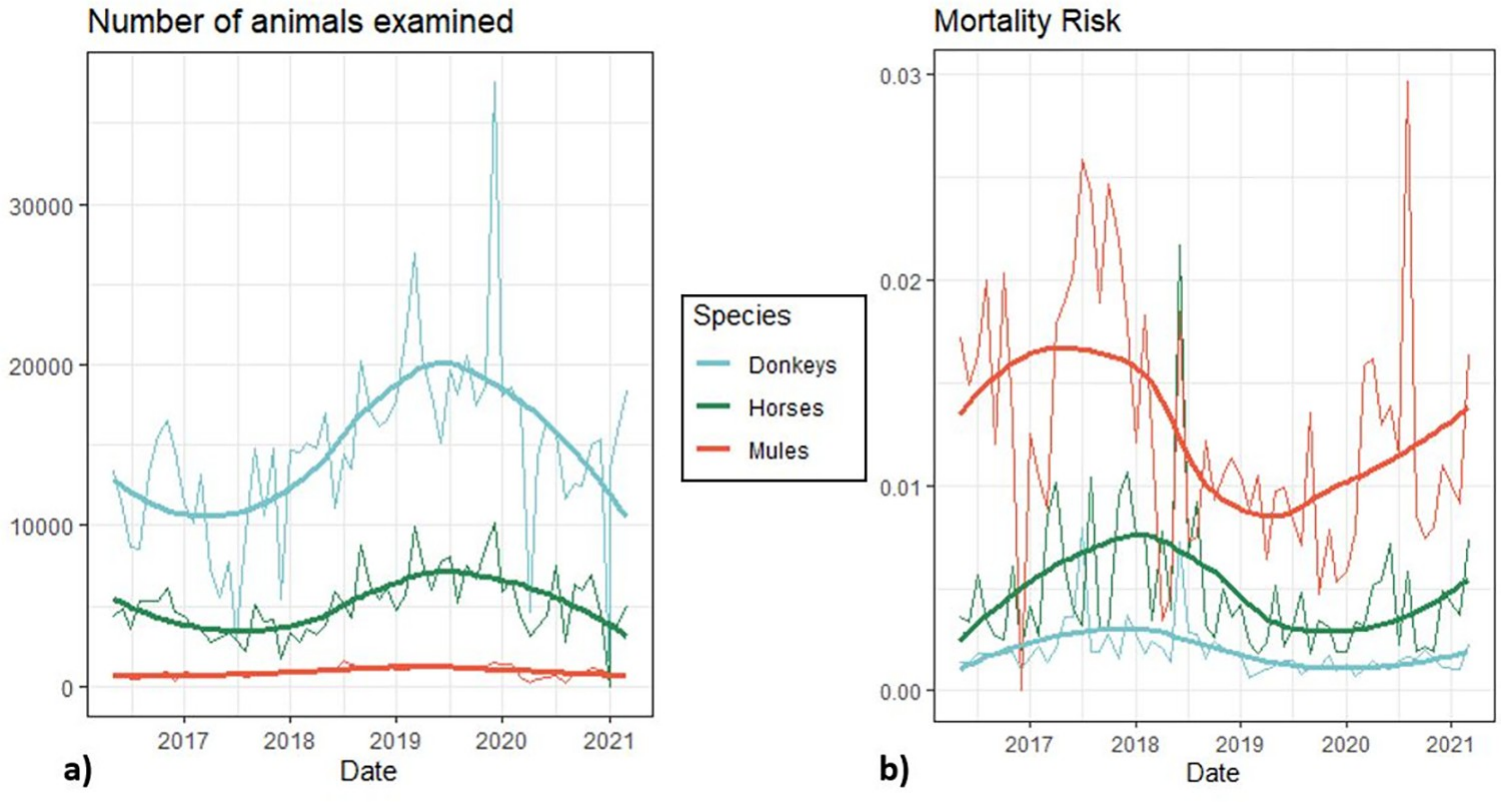
Number of animals seen per species per year in all countries included in retrospective data analysis of clinical findings of working equids in low- and middle-income countries presenting to an international NGO between May 2016 and March 2021 (a)); and mortality risk (proportion) of species in relation to the overall number of animals presented per species per year (b)). An outlier in horse mortality risk during January 2021 has been removed from plot b) for visualization purposes. Solid curve lines generated with geom_smooth() function and LOESS smoother method in R 4.2.1.

The highest frequency of equid mortalities was reported in donkeys (49.8%; 4,993/10,018), followed by horses (32.1%; 3,219/10,018) and mules (18%; 1,806/10,018). During May 2016 to March 2021 the overall mortality risk (proportion of species mortality in relation to the number of animals of the same species examined) of mules was 12 deaths per 1,000 animals (95% CI 9–15), 4 deaths per 1,000 animals (95% CI 4–5) in horses and 2 deaths per 1,000 animals (95% CI 1–2) in donkeys. Mortality risk differed between species, with mules having a 6.7 times higher mortality risk compared to donkeys (95% CI 4.91–9.02; p<0.001) and a 2.7 times higher mortality risk compared to horses (95% CI 1.96–3.61; p<0.001) over the same period of time. In turn, horses had a 2.5 times higher mortality risk than donkeys (95% CI 1.86–3.37; p<0.001) (Fig 2(B)).

### Clinical findings

**Fig 3:**
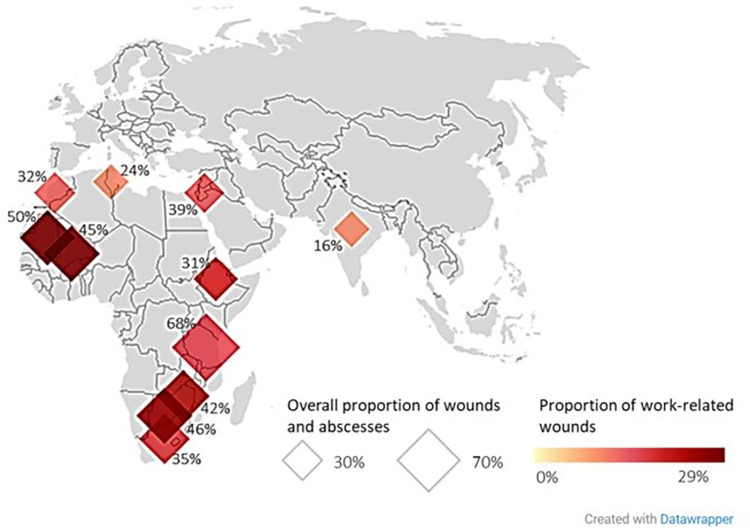
Overall proportion of wounds and abscesses and proportion of work-related wounds for each country included in retrospective data analysis of clinical findings of working equids in low- and middle-income countries presenting to an international NGO between January 2005 and March 2021. The overall proportion of wounds and abscesses in relation to all other clinical findings (category 1) is represented by diamond size and value, and the proportion of specifically work-related wounds (findings category 2) in relation to all other clinical findings is represented by diamond colour (darker colours indicating higher proportion of work-related wounds). Figure created in Datawrapper [21].

Wounds and abscesses were the most common category of clinical findings (mean monthly proportion 35% ±19%) recorded during the study period in all species of equids regardless of categorization used, and work-related wounds were the predominant type of wound (mean proportion 17.8%; ±16%) (Fig 3). The mean monthly proportions of clinical findings by categories 1 and 2 are summarised in Table 1 and S4 File.

Using findings category 1, wounds and abscesses represented the highest proportion of clinical findings in all countries except Kenya, South Africa, India and Algeria (S4 File). When considering findings category 2, musculoskeletal problems were the predominant type of clinical finding in Jordan (19.6%; ±0.12) and Morocco (17.5%; ±0.04), parasitism in Tunisia (34.5%; ±0.09), South Africa (32.8%; ±0.37) and Zimbabwe (21.2%; ±0.21), and non-parasitic infectious diseases were the predominant clinical finding in Ethiopia (30.4%; ±0.21) (S4 File).

**Fig 4:**
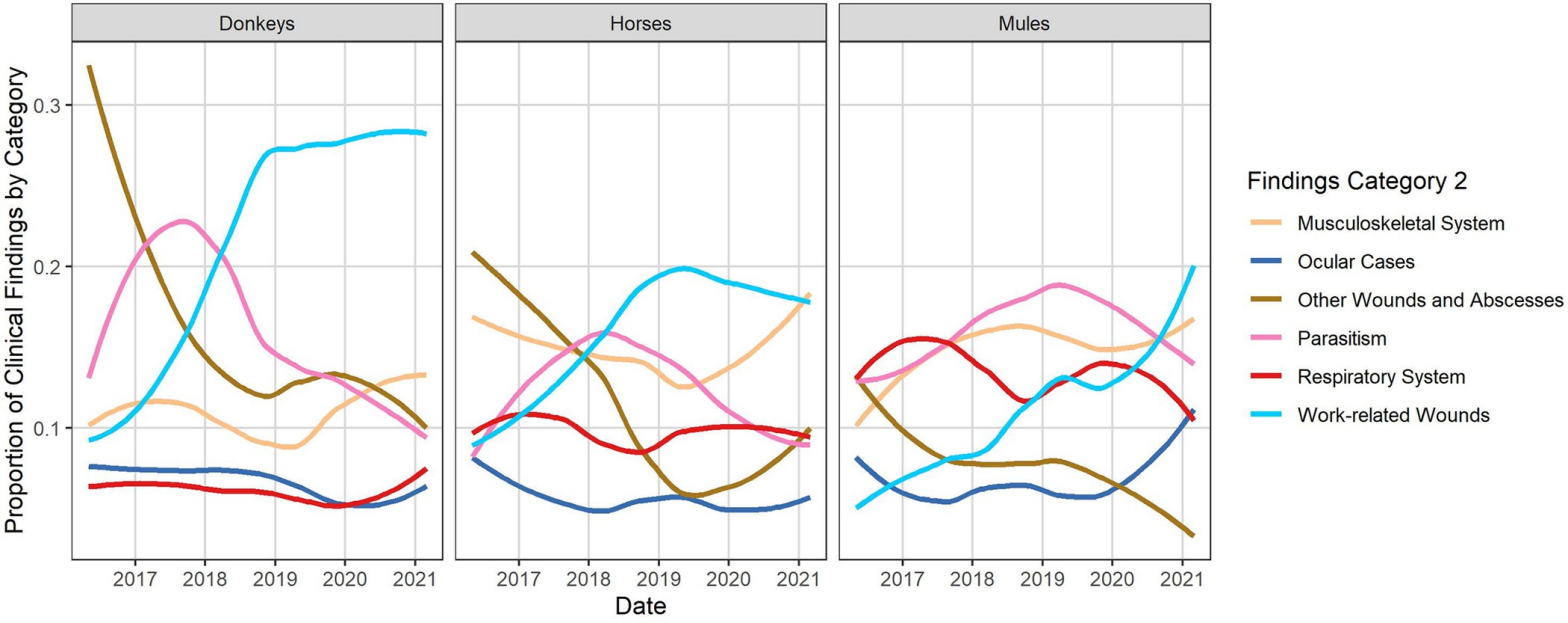
Proportion of the six most frequent categories of clinical findings by species and date, using Findings Category 2, in a retrospective study of clinical findings of working equids in low- and middle-income countries presenting to an international NGO between May 2016 and March 2021. Solid curve lines generated with geom_smooth() function and LOESS smoother method in R 4.2.1.

Wounds and abscesses (Findings Category 1) were reported as the highest proportion of clinical findings for all species (donkeys 44.7%, 400,279/895,777; horses 32.7%, 198,081/605,197; mules 31.4% 92,732/294,903), with an overall proportion of 38.5% (691,092/1,795,877). Using Findings Category 2, work-related wounds were the predominant finding in donkeys (22.1%; ±19%) and horses (16%; ±14%), and parasitism was the most common in mules (16%; ±20%) (Fig 4 and S4 File).

**Fig 5:**
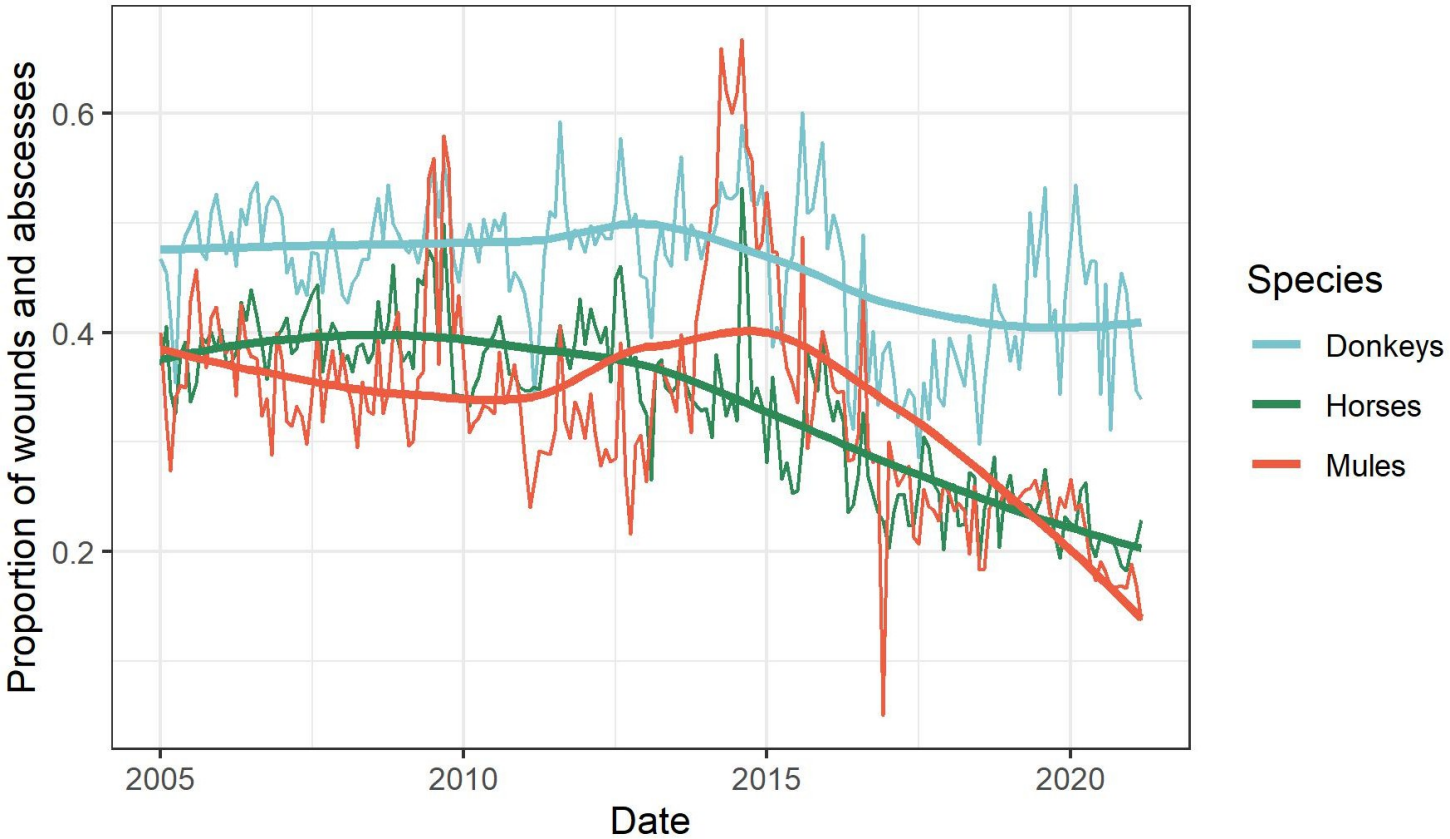
Proportion of wounds and abscesses (Findings Category 1) in relation to all other clinical findings by species and date in a retrospective study of clinical findings of working equids in low- and middle-income countries presenting to an international NGO between January 2005 and March 2021. Solid curve lines generated with geom_smooth() function and LOESS smoother method in R 4.2.1.

When adjusting for changes in time and reporting template, donkeys presented with a higher proportion of wounds and abscesses in relation to other finding categories than mules (RR 1.49; 95% CI 1.37–1.61; p<0.001) or horses (RR 1.45; 95% CI 1.34–1.58; p<0.001) (Fig 5 and S5 File).

The mean proportion of wounds and abscesses per calendar month ranged from 35.7% (±9%) in March to 41.8% (±11%) in August, with no significant difference in proportion of wounds per calendar month. The overall proportion of wounds and abscesses (findings category 1) in relation to other clinical findings decreased over time (coef. -0.23 per 100 months on natural log scale; 95% CI -0.39 to -0.07; p = 0.004), although no significant decrease was seen when adjusting for change in reporting template. The proportion of ocular-aural findings (coef. -0.27 per 100 months on natural log scale; 95% CI -0.42 to -0.12; p = 0.033) also decreased over the study period when adjusted for changes in reporting template.

## Discussion

This retrospective study analyses over one million clinical findings of working equids in LMICs presenting to an international NGO across 14 countries and over a 16-year period. In this study, wounds were the most reported type of clinical findings in all equid species. Donkeys presented with a disproportionately higher amount of wounds in relation to other equid species while mortality risk in mules was higher than in donkeys or horses. Parasitism was another commonly reported clinical finding. Although data format is critical for disease surveillance, datasets from international NGOs can provide an important epidemiological insight into the health of these animals.

This study identified an overall mortality risk of 3 deaths per 1,000 animals across all equid species between May 2016 and March 2021. Studies on other general equine populations in Europe report from 18 to more than 40 all-cause deaths per 1,000 animals [22, 23]. As diseases with high prevalence, morbidity and mortality risk are common among working equids [24–26] and these animals often have lower life expectancy [27, 28] than other groups of equids, a higher mortality risk might therefore be expected. In the current study, mortality risk may be under-reported due to a lack of patient follow-up associated with the type of single-contact interventions practiced in most mobile clinics. Additionally, under-reporting may reflect a bias where mortality and euthanasia are sensitive topics within specific cultural settings or NGO project and operational aims. In many countries, euthanasia is not a culturally or religiously acceptable practice and mortality is likely to occur mostly outside the veterinary setting and therefore not be captured within health records. Nonetheless, this highlights a need for better reporting and understanding of morbidity and mortality in working equid populations. Although mules represented the smallest proportion of animals presenting to veterinary clinics in relation to other equid species, they had a higher mortality risk than donkeys or horses. There are few studies relating to the pathology and mortality of working mules. While Ali et al. [29] suggest mules may be physically better suited than donkeys to work environments such as brick kilns, other reports suggest that required physiological adaptation of mules to challenging environments may be associated with mortality [30]. The often difficult and aggressive temperament of mules [29, 30] could also help to explain a higher mortality, potentially making these animals less amenable to nurse or keep if injured. Finally, a higher mule mortality may be due to the specific context or differences in reporting across countries that report mule presentations. Nevertheless, further research is warranted into mule health and working equid mortality given the scarcity of literature on this topic.

Other studies also report high frequencies of wounds and skin lesions in working equids [9, 10, 12, 31–39], particularly work-related wounds [9, 31, 34–39]. Despite being well documented, the high prevalence of work-related wounds remains a particular welfare concern for working equids, to which they are predisposed to by the very nature of their work. This highlights the importance of appropriate work practices and equipment, such as harness or pack design, cleanliness and materials [31, 34, 38–40], in a holistic approach to wound prevention which should remain at the centre of efforts to safeguard the welfare of these animals. Analysing the incidence of work-related wounds over time, through changes in reporting format, would be a useful indicator of owner behaviour change and the organisation’s impact on work practices through education, preventive measures and distribution of equipment.

A higher proportion of wounds was recorded in donkeys in relation to any other category of findings than in mules or horses, albeit with a modest relative risk. Other studies have also found donkeys to have the highest prevalence of wounds compared to other equids [9, 31], although this is not consistently reported with mules [10, 12] or horses [37, 41] having higher wound prevalence in some studies. This could suggest wounds are indeed more frequent in donkeys, potentially due to the draught or pack work they typically perform being associated with higher prevalence of wounds than other work types [9, 10], or due to inadequate work practices such as equipment condition, padding and cleanliness [31, 34, 38]. Donkeys are also typically the least costly working animal to purchase and maintain and often perceived as having the lowest value [42, 43]. This may contribute to higher pressures or reduced care, potentially translating into donkey’s frequently poorer welfare [9], although owner socioeconomic factors don’t necessarily correlate to animal welfare status [44]. On the other hand, the predominance of wounds in relation to other clinical findings could be associated with difficulty diagnosing other types of conditions in this species. Donkeys are often perceived as stoic and identifying objective indicators of pain can be more challenging in this species [45, 46]. As such, other conditions or manifestations of pain might not be as evident to owners or carers to prompt presentation to a veterinary clinic [45, 47, 48]. Additionally, despite clinical presentations and survival outcomes of donkey conditions often not being directly comparable to those of horses [46, 49, 50] due to physiological and behavioural differences [50], equid veterinary teaching tends to be focused on horses, potentially making other diagnoses in donkeys harder.

Parasitism was the second most reported category of clinical findings after wounds at overall study level. While there is limited information on the epidemiology of parasitic infections in working equids of most African countries [51, 52], this raises important considerations regarding parasitic burdens, diagnostic accuracy and appropriateness of therapeutic approaches. It is not possible to infer diagnostic methods, treatment prescribed or resistance levels from this dataset, and no differentiation between types of parasites was possible in this study. However, logistical issues associated with the large numbers of animals seen at ambulatory settings and an often-limited diagnostic capacity pose serious yet frequently unavoidable challenges to confirmation of parasitic infection. As such, strategic deworming becomes increasingly important, understanding that a degree of parasitic burden does not necessarily result in clinical disease and the risk of parasitic infection varies according to various population and management factors [53]. Concerns for anthelmintic resistance in equids are growing [54–57], particularly in face of global warming [58, 59], and some studies suggest a reduced efficacy of anthelmintics in populations of African donkeys [52, 60]. Blanket deworming approaches are considered inadequate and ineffective in some cases [53, 61], and may represent an unnecessary cost to those already under severe economic pressure [51, 62] and pose a significant risk to the development of anthelmintic resistance [53, 63]. As such, given the frequency of parasitic cases reported, strategic and accountable veterinary practices with responsible use of anthelmintics [55], as well as surveillance of parasitic burdens and emergence of resistance are therefore critical to prevent fostering resistance among this population of animals.

Few changes over time in reporting of clinical findings were observed. While this could mean a failure of educational efforts or inefficient preventive strategies, it is more likely a reflection of the organisational strategy to operate in the areas of greatest need, where clinic locations within each country may change over time to serve the most at need populations once progress has been made elsewhere. Similarly, measurement bias may be present due to variations in clinical acumen or capacity, where an increase in the proportion of certain categories could be related to increased availability of diagnostic capacity, experience, tools or training, leading for example to a higher proportion or oral findings once dentistry tools or training are provided to that project. Therefore, recording the prevalence and incidence of clinical findings at individual animal level would enable a more meaningful interpretation of population health status and monitor trends of preventable conditions over time. Further investigation of changes in type of intervention across different countries and how this translates into animal health indicators would provide valuable operational insight and could reflect an epidemiologic transition in the health status of each population. Similarly, it would be appropriate to use the countries that did not report on wounds as the predominant type of clinical finding as case studies to understand potential risk and protective factors for wounds. Fluctuations or differences associated with seasonal infectious diseases, agricultural work intensity, climate events or reduced feed availability may also be present and understanding them would help devise appropriate prevention and response strategies.

Working equids of several LMICs are frequently not recognised as an individual group of animals nor are they necessarily considered within livestock interventions, which means they are often not captured by the animal health surveillance systems of structures they are inserted in [4, 64]. Although there is reasonably good evidence of the pathogenesis of common diseases and welfare issues, there is a concerning lack of epidemiological understanding of the prevalence, behaviour and transmission of working equid conditions [64, 65], including infectious and zoonotic risks [65], which poses a threat to the One Health of communities. The lack of appropriate working equid disease surveillance is complex and multifactorial, but it can in part be attributed to significant technical data gaps [64], often due to reduced government capacity in LMICs [66]. Such data would be invaluable not only for disease surveillance but also for policy development, research and public health monitoring. The fact that some findings from this study such as the high proportion of wounds, parasitism or musculoskeletal findings are in line with results of purpose-built research studies [10, 12, 33, 36], highlights the potential of using routine monitoring data from organisations on the ground for animal health surveillance purposes. However, an understanding of disease incidence and prevalence by identifying the proportion of individual animals with specific conditions (numerator) within a population of animals (denominator) would be required for adequate veterinary surveillance. Data collection format should therefore enable this, providing individual level tracing of animals through animal identification which can be particularly challenging.

One of the main limitations of this retrospective study is that it relies on data recorded and reported by many individuals across multiple clinics, partner organisations, and countries. The likelihood of owners presenting to the NGO’s clinics may vary according to the availability of alternative veterinary services and the relationship between the organization and the local communities, which represents a potential bias. Although reporting guidelines cannot ensure consistency of reporting which may be affected by country differences in recording, diagnostic accuracy or NGO resources, analysing this dataset at findings category level rather than individual clinical findings reduces this potential bias. Due to the volume of animals seen, the nature of the NGO clinical work and the context in which they operate, selection bias due to under or incomplete reporting may occur and there is no individual-level tracing of animals. As such, an understanding of incidence and prevalence is not possible. Additionally, the same animal may contribute multiple clinical findings. The severity of findings is also not represented, and it is therefore possible that categories observed in lower proportion may have a higher impact on morbidity than those observed in higher proportion. Nevertheless, investigating the combined findings of animals seen by LMIC veterinary services over 15 years provides an important overview of the predominant health concerns of working equids across countries and species, and analysing them as a proportion within all reported findings allows a good understanding of their relative significance. Dates and months are likely to be serially correlated which may influence the overall goodness of fit of the multivariable binomial regression models. However, AR(1) autocorrelation of error terms within each species, calendar month and hemisphere were incorporated in the models to account for this and minimize this limitation. Finally, these results refer to specific geographical locations and cannot be extrapolated to the national equid population of included countries. Nevertheless, they provide valuable, epidemiological insight into regional working equid conditions.

## Conclusions and recommendations

This study investigates clinical findings of working equids, their spatial and temporal distributions, and epidemiological patterns. With a view to reducing the high frequency of work-related wounds, strategies to prevent wounds in working equids should focus on work equipment as well as work and husbandry practices. We found that donkeys had disproportionally more wounds than other species, while mortality risk was higher in mules than other equids. Awareness and training of owners and veterinary professionals on donkey and mule welfare is therefore recommended, as well as further investigation of causes of working equid mortality. This is a rich dataset that warrants further investigations of variation in clinical findings and mortality at a more granular level, according to season and geographic region. Routine monitoring data from international NGOs could be included in longitudinal studies and animal health surveillance strategies, although appropriate data formats are critical to enable this. Standardized reporting with individual level tracing of animals to allow an understanding of prevalence, animal and disease recurrence, as well as risk factors for clinical findings is recommended to enable more meaningful epidemiological surveillance.

## Supporting information

S1 File Re-categorization of clinical findings and finding categories. (XLSX)

S2 File Inclusivity in global research questionnaire. (DOCX)

S3 File Number of equids and presentations. (DOCX)

S4 File Analysis of clinical findings. (DOCX)

S5 File Statistical modelling plots. (DOCX)
