# Supplementary material for: Common clinical findings identified in working equids in low- and middle-income countries from 2005 to 2021
Source: PLoS One. 2024 Jun 5;19(6):e0304755. doi: 10.1371/journal.pone.0304755 (PMC11152255; doi:10.1371/journal.pone.0304755)
Supplement: S3 File — (DOCX) [file pone.0304755.s003.docx]

**Supplement 3. Number of clinical findings, treatments, equipment distributions and animals per species per year.**

Supplement 3 Table 1 - Year and species breakdown of available data for number of working equid clinical findings, treatments, equipment distribution and number of animals seen by an international NGO between January 2005 March 2021

| **Year** | Horse clinical findings, treatments & equipment distribution | | Donkey clinical findings, treatments & equipment distribution | Mule clinical findings, treatments & equipment distribution | **Total clinical findings, treatments & equipment distribution** | Horses Examined | Donkeys Examined | Mules Examined | **Total Equids Examined** | Horse Deaths | Donkey Deaths | Mule Deaths | **Total Equids Deaths** |
| --- | --- | --- | --- | --- | --- | --- | --- | --- | --- | --- | --- | --- | --- |
| **2005** | 88,523 | | 120,300 | 45,579 | **254,402** |  |  |  |  | 81 | 316 | 69 | **466** |
| **2006** | 61,654 | | 120,154 | 30,362 | **212,170** |  |  |  |  | 94 | 239 | 98 | **431** |
| **2007** | 79,996 | | 148,771 | 54,999 | **283,766** |  |  |  |  | 196 | 460 | 162 | **818** |
| **2008** | 73,926 | | 128,840 | 52,762 | **255,528** |  |  |  |  | 153 | 270 | 104 | **527** |
| **2009** | 73,832 | | 128,455 | 46,995 | **249,282** |  |  |  |  | 173 | 234 | 122 | **529** |
| **2010** | 81,153 | | 138,648 | 40,703 | **260,504** |  |  |  |  | 216 | 323 | 273 | **812** |
| **2011** | 85,820 | | 155,433 | 51,826 | **293,079** |  |  |  |  | 249 | 474 | 78 | **801** |
| **2012** | 79,098 | | 148,103 | 43,512 | **270,713** |  |  |  |  | 224 | 388 | 114 | **726** |
| **2013** | 51,554 | | 93,123 | 24,842 | **169,519** |  |  |  |  | 178 | 304 | 73 | **555** |
| **2014** | 42,902 | | 114,942 | 22,381 | **180,225** |  |  |  |  | 159 | 316 | 65 | **540** |
| **2015** | 38,673 | | 124,525 | 11,747 | **174,945** |  |  |  |  | 129 | 161 | 35 | **325** |
| **2016** | 64,200 | | 163,040 | 31,549 | **258,789** | 39,176* | 101,701* | 5,191* | **146,068*** | 193 (143*) | 201 (164*) | 92 (77*) | **486 (384*)** |
| **2017** | 63,260 | | 203,710 | 25,355 | **292,325** | 40,535 | 113,318 | 8,817 | **162,670** | 232 | 264 | 160 | **656** |
| **2018** | 78,399 | | 232,936 | 35,782 | **347,117** | 58,449 | 185,473 | 12,695 | **256,617** | 392 | 427 | 122 | **941** |
| **2019** | 92,115 | | 290,123 | 35,794 | **418,032** | 87,900 | 252,243 | 14,571 | **354,714** | 249 | 292 | 123 | **664** |
| **2020** | 73,146 | | 205,039 | 24,346 | **302,531** | 61,919 | 172,381 | 9,313 | **243,613** | 207 | 225 | 93 | **525** |
| **2021** (Jan-March) | 16,332 | | 69,570 | 4,777 | 90,679 | 12,701 | 47,789 | 2,240 | **62,730** | 94 | 99 | 23 | **216** |
| **TOTAL** | **1,144,583** | | **2,585,712** | **583,311** | **4,313,606** | 300,680 | 872,905 | 52,827 | **1,226,412** | **3,219 (1,317*)** | **4,993 (1,471*)** | **1,806 (598*)** | **10,018 (3,386*)** |
| *Data available or analyzed from May 2016 onwards | | | | | | | | | | | |  |  |
|  | | | | | | | | | | | |  |  |


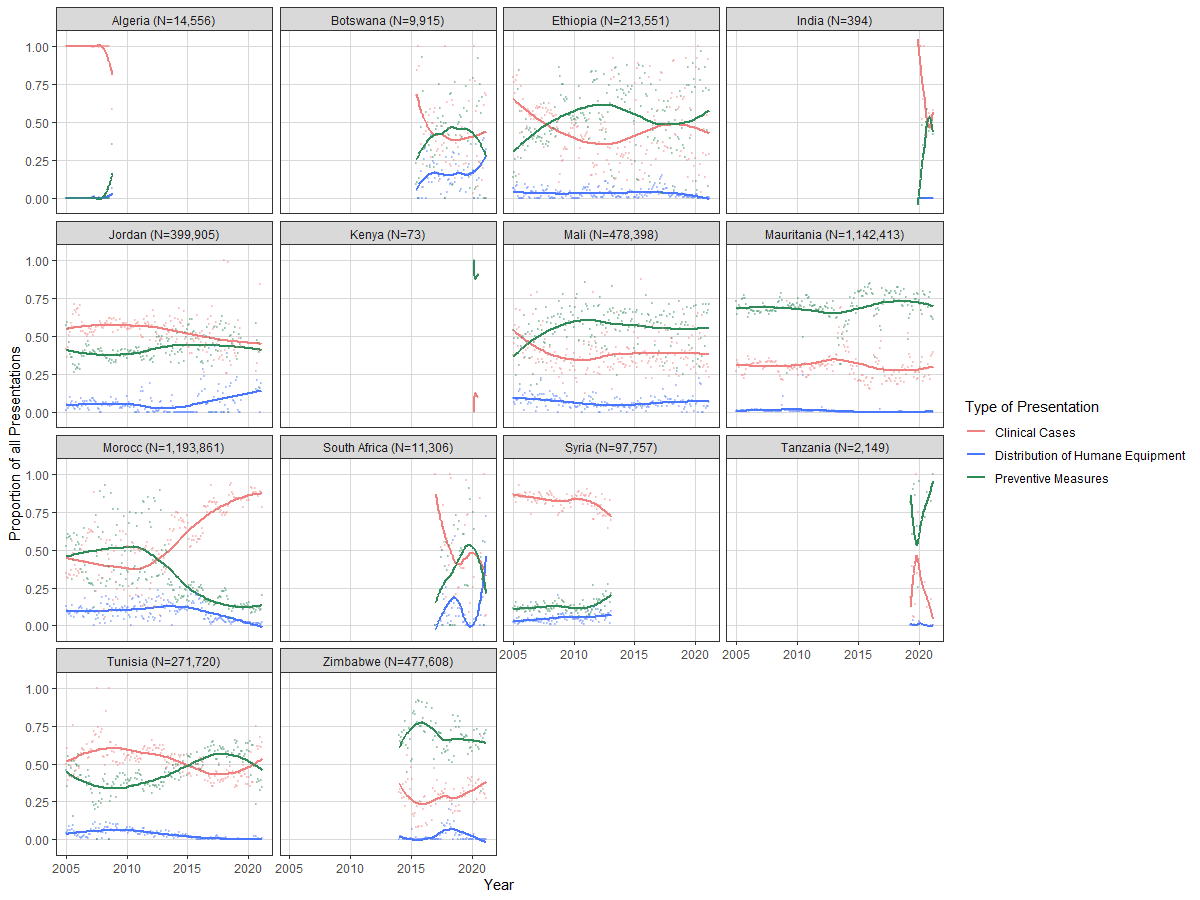
Supplement 3 Figure 1 – Proportion of each type of veterinary intervention provided to working equids presenting to clinics of an international NGO in 14 countries during January 2005 and March 2021. The frequency of presentations (clinical findings, treatments and equipment distribution) per country during the study period is provided. Frequencies represent number of presentations only and do not account for number of animals. Solid curve lines generated with geom_smooth() function and LOESS smoother method in R 4.2.1.

**
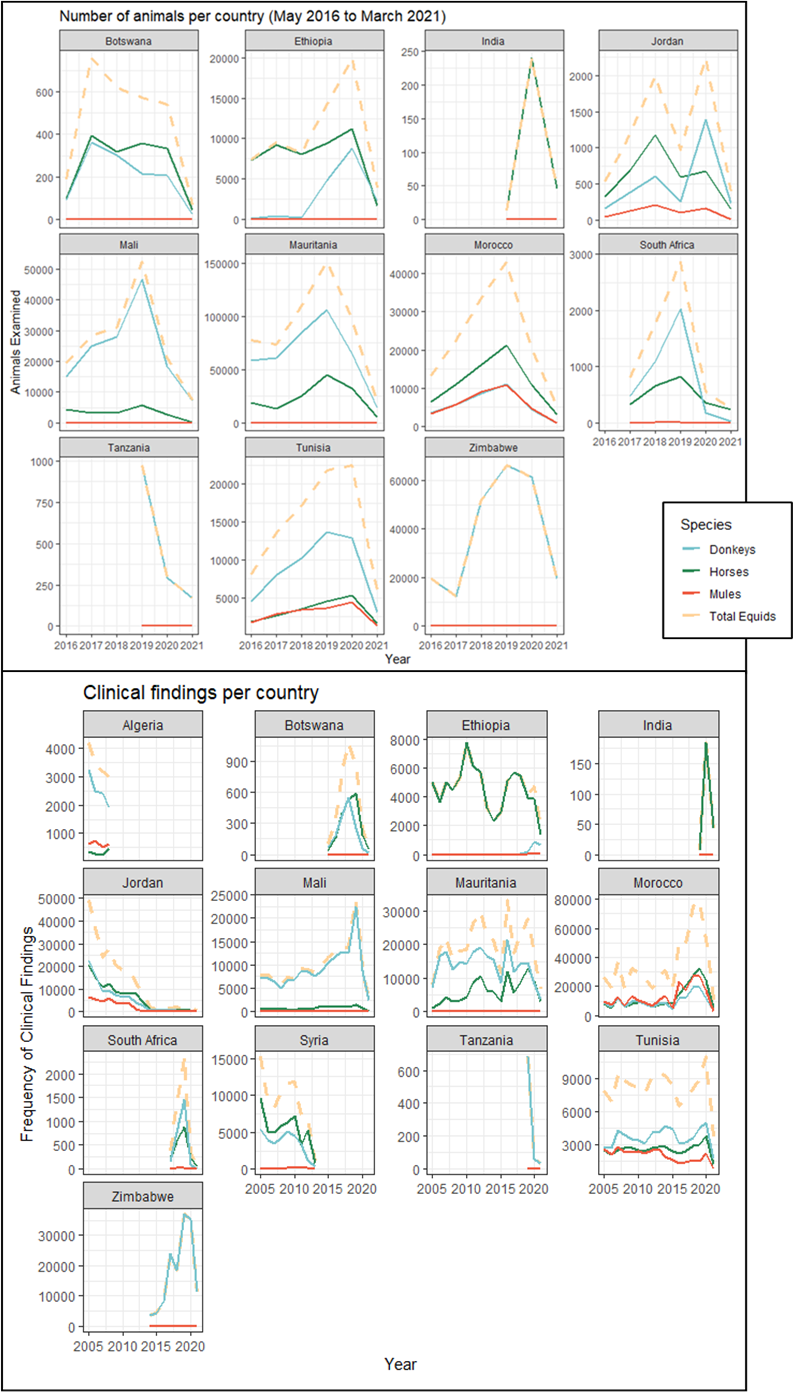
**

Supplement 3 Figure 2 – Total number of animals examined between May 2016 and March 2021 (top) and frequency of clinical findings (bottom) during the study period according to country and equid species included in retrospective data analysis of clinical findings of working equids in low- and middle-income countries presenting to an international NGO between 2005 and 2021.

*Supplement 3 Table 2 - Overall mortality rate per year and country* *included in retrospective data analysis of clinical findings of working equids in low- and middle-income countries presenting to an international NGO between May 2016 to March 2021 only.*

**Mortality Rates per Year and Country**

| Year Totals | Total Equids Examined | Total Equid Mortality | Mortality Risk |
| --- | --- | --- | --- |
| 2016  (May-December) | 146,068 | 384 | 0.26% |
| 2017 | 162,670 | 656 | 0.40% |
| 2018 | 256,617 | 941 | 0.37% |
| 2019 | 354,714 | 664 | 0.19% |
| 2020 | 243,613 | 525 | 0.22% |
| 2021  (January-March) | 62,730 | 216 | 0.34% |
|  |  |  |  |
| Country Totals |  |  |  |
| Botswana | 2,744 | 102 | 3.72% |
| Morocco | 137,829 | 2,043 | 1.48% |
| Ethiopia | 60,962 | 405 | 0.66% |
| South Africa | 6,229 | 28 | 0.45% |
| Jordan | 7,360 | 25 | 0.34% |
| Zimbabwe | 231,085 | 247 | 0.11% |
| Mali | 159,468 | 126 | 0.08% |
| Mauritania | 529,826 | 356 | 0.07% |
| Tunisia | 89,067 | 54 | 0.06% |
| India | 297 | 0 | 0% |
| Kenya | 109 | 0 | 0% |
| Tanzania | 1,436 | 0 | 0% |


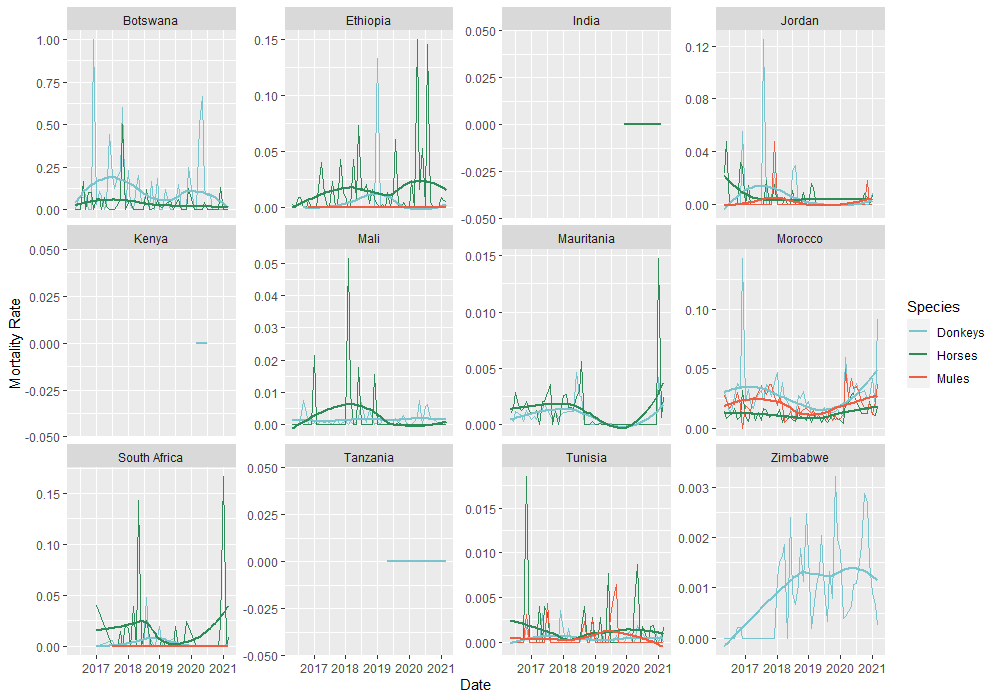


Supplement 3 Figure 3 - Mortality rates (as a proportion) according to country, equid species and year, for 12 countries with veterinary projects attending working animals between May 2016 and March 2021. Solid curve lines generated with geom_smooth() function and LOESS smoother method in R 4.2.1.
