# Supplementary material for: Common clinical findings identified in working equids in low- and middle-income countries from 2005 to 2021
Source: PLoS One. 2024 Jun 5;19(6):e0304755. doi: 10.1371/journal.pone.0304755 (PMC11152255; doi:10.1371/journal.pone.0304755)
Supplement: S4 File — (DOCX) [file pone.0304755.s004.docx]

**Supplement 4. Analysis of the proportion of clinical findings per country and per year.**

Supplement 4 Table 1 - Mean proportion and standard deviation of clinical findings by category in relation to all other finding categories per species, for all countries in a retrospective study of clinical findings of working equids in low- and middle-income countries presenting to an international NGO between January 2005 and March 2021. Two clinical finding categorisations used and describes. Syndrome category 2 was analysed from May 2016 to March 2021 only.

**Proportion of clinical findings per species**

| Findings Category 1 | | | | | | | | | | |  | Findings Category 2  *May 2016 to Mar 2021 only | | | | | | | | | |
| --- | --- | --- | --- | --- | --- | --- | --- | --- | --- | --- | --- | --- | --- | --- | --- | --- | --- | --- | --- | --- | --- |
|  | **Horses** | | | **Donkeys** | | | **Mules** | | | |  |  | | **Horses** | | | **Donkeys** | | | **Mules** | |
|  | Proportion ±SD | | Proportion ±SD | | | Proportion ±SD | | |  |  | | | Proportion ±SD | | | Proportion ±SD | | | Proportion ±SD | | |
| Wounds and Abscesses | 32.1% | ±0.16 | 41.7% | | ±0.20 | 28.3% | | ±0.19 |  | Work-related Wounds | | | 16.0% | | ±0.14 | 22.1% | | ±0.19 | 10.9% | | ±0.12 |
| Other Medical Cases | 16.8% | ±0.19 | 16.1% | | ±0.19 | 17.2% | | ±0.25 |  | Parasitism | | | 12.4% | | ±0.17 | 16.1% | | ±0.20 | 16.0% | | ±0.20 |
| Musculoskeletal System | 13.6% | ±0.08 | 9.9% | | ±0.09 | 12.2% | | 0.11 |  | Musculoskeletal System | | | 14.9% | | ±0.11 | 11.2% | | ±0.12 | 14.9% | | ±0.09 |
| Respiratory System | 11.4% | ±0.09 | 7.3% | | ±0.07 | 12.4% | | ±0.12 |  | Other Wounds and Abscesses | | | 11.4% | | ±0.12 | 15.4% | | ±0.17 | 7.7% | | ±0.10 |
| Skin Complaints | 9.4% | ±0.12 | 9.4% | | ±0.13 | 9.7% | | ±0.15 |  | Respiratory System | | | 9.9% | | ±0.10 | 6.1% | | ±0.06 | 13.7% | | ±0.10 |
| Digestive System | 7.0% | ±0.06 | 5.4% | | ±0.05 | 7.8% | | ±0.10 |  | Ocular Cases | | | 5.6% | | ±0.06 | 6.6% | | ±0.08 | 6.6% | | ±0.07 |
| Ocular and Aural Cases | 5.2% | ±0.05 | 6.4% | | ±0.07 | 5.2% | | ±0.07 |  | Other Medical Cases | | | 6.4% | | ±0.10 | 5.8% | | ±0.10 | 5.0% | | ±0.11 |
| Oral Problems | 3.1% | ±0.06 | 2.5% | | ±0.05 | 5.3% | | ±0.12 |  | Oral Problems | | | 5.2% | | ±0.07 | 4.3% | | ±0.07 | 11.8% | | ±0.17 |
| Surgical Cases | 1.4% | ±0.05 | 1.4% | | ±0.06 | 1.8% | | ±0.12 |  | Digestive System | | | 6.7% | | ±0.08 | 4.9% | | ±0.06 | 5.7% | | ±0.05 |
| Cardiovascular System | 0.9% | ±0.03 | 0.8% | | ±0.02 | 1.4% | | ±0.05 |  | Non-parasitic Infectious Diseases | | | 7.5% | | ±0.13 | 2.8% | | ±0.07 | 4.3% | | ±0.14 |
| Road Traffic Accidents | 0.7% | ±0.02 | 0.8% | | ±0.03 | 0.1% | | ±0.00 |  | Skin Complaints | | | 1.7% | | ±0.04 | 2.3% | | ±0.07 | 0.7% | | ±0.01 |
|  |  |  |  | |  |  | |  |  | Surgical Cases | | | 1.4% | | ±0.05 | 1.4% | | ±0.06 | 1.8% | | ±0.12 |
|  |  |  |  | |  |  | |  |  | Cardiovascular System | | | 0.8% | | ±0.03 | 0.8% | | ±0.03 | 1.2% | | ±0.05 |
|  |  |  |  | |  |  | |  |  | Road Traffic Accidents | | | 0.7% | | ±0.02 | 0.8% | | ±0.03 | 0.1% | | ±0.00 |
|  |  |  |  | |  |  | |  |  | Aural Problems | | | 0.2% | | ±0.00 | 0.3% | | ±0.01 | 0.1% | | ±0.00 |

| 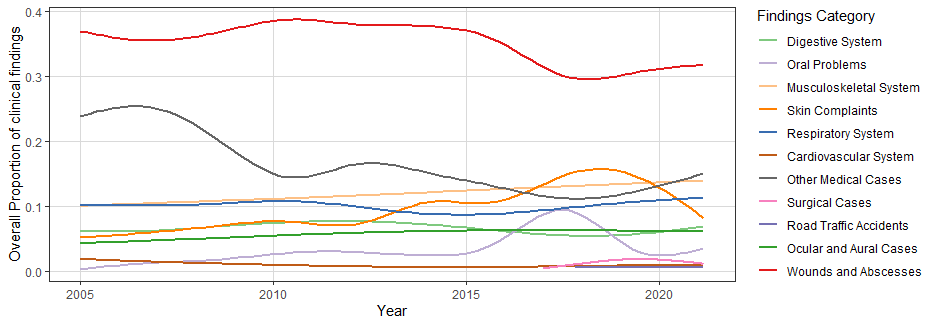 |
| --- |
| 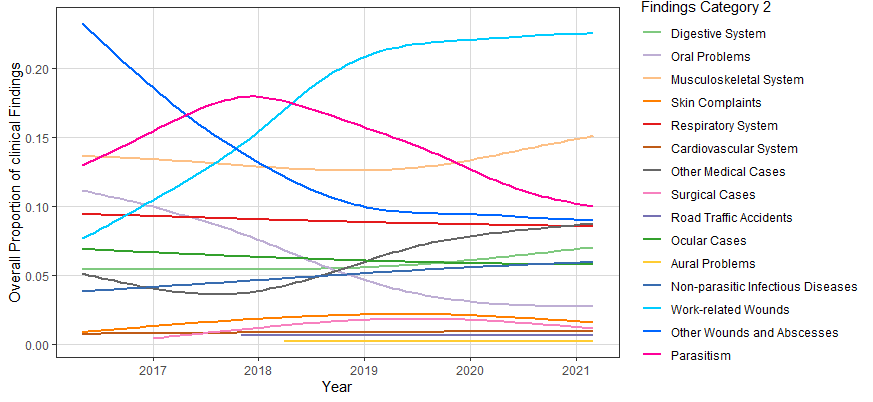 |

Supplement 4 Figure 1 - Overall proportion of clinical findings by category for all countries using findings category 1 (top) in a retrospective study of clinical findings of working equids in low- and middle-income countries presenting to an international NGO between January 2005 and March 2021. Overall proportion of clinical finding categories for all countries between May 2016 and March 2021 using findings category 2 (bottom). Solid curve lines generated with geom_smooth() function and LOESS smoother method in R 4.2.1.


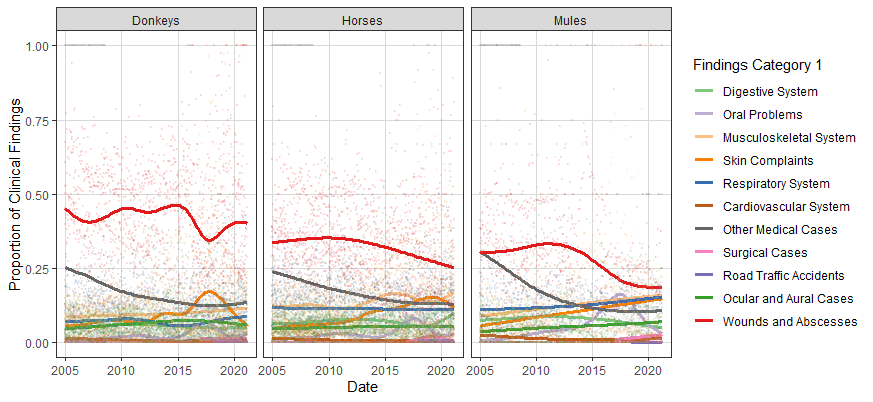


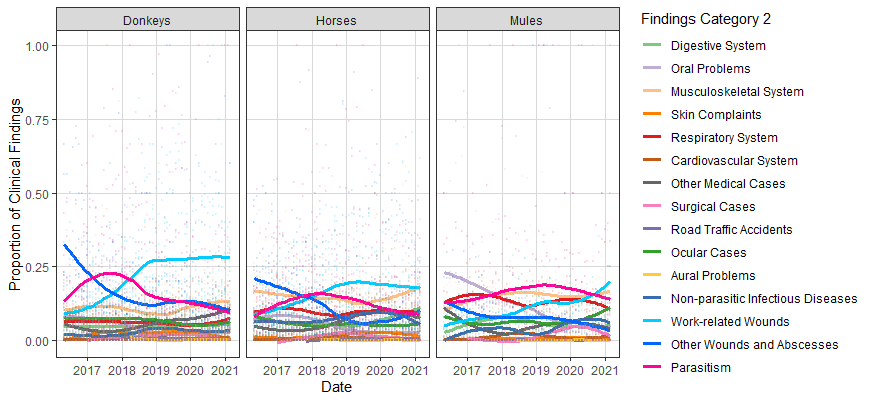


Supplement 4 Figure 1.1 - Overall proportion of the 6 most common clinical finding categories by species using category 1 (top) in a retrospective study of clinical findings of working equids in low- and middle-income countries presenting to an international NGO between January 2005 and March 2021; and overall proportion of the 6 most common clinical finding categories by species between May 2016 and March 2021 using an alternative categorisation (findings category 2) (bottom). Solid curve lines generated with geom_smooth() function and LOESS smoother method in R 4.2.1.

| 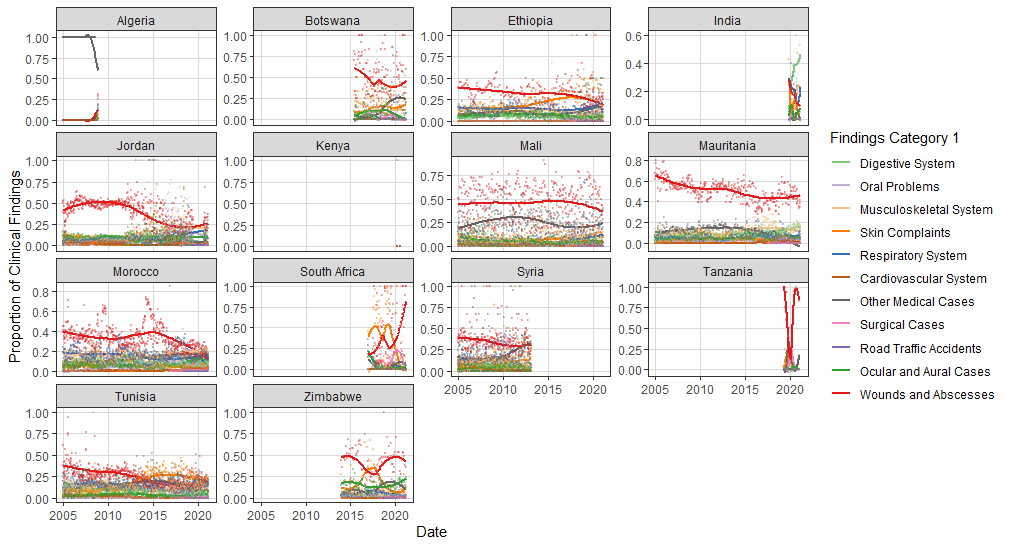 |
| --- |
| 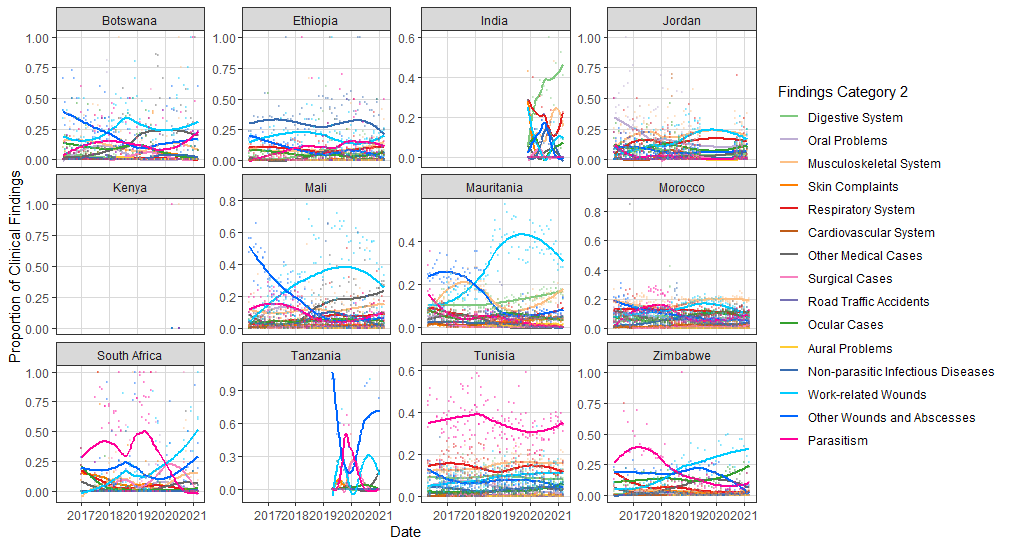 |

Supplement 4 Figure 2 - Overall proportion of clinical findings (all equids) per country using findings category 1 (top) in a retrospective study of clinical findings of working equids in low- and middle-income countries presenting to an international NGO between January 2005 and March 2021. Overall proportion of clinical findings (all equids) per country between May 2016 and March 2021 using findings category 2 (bottom). Solid curve lines generated with geom_smooth() function and LOESS smoother method in R 4.2.1.
