# Supplementary material for: Common clinical findings identified in working equids in low- and middle-income countries from 2005 to 2021
Source: PLoS One. 2024 Jun 5;19(6):e0304755. doi: 10.1371/journal.pone.0304755 (PMC11152255; doi:10.1371/journal.pone.0304755)
Supplement: S5 File — (DOCX) [file pone.0304755.s005.docx]

**Supplement 5. Statistical modelling of the proportion of wounds and mortality risk per species**


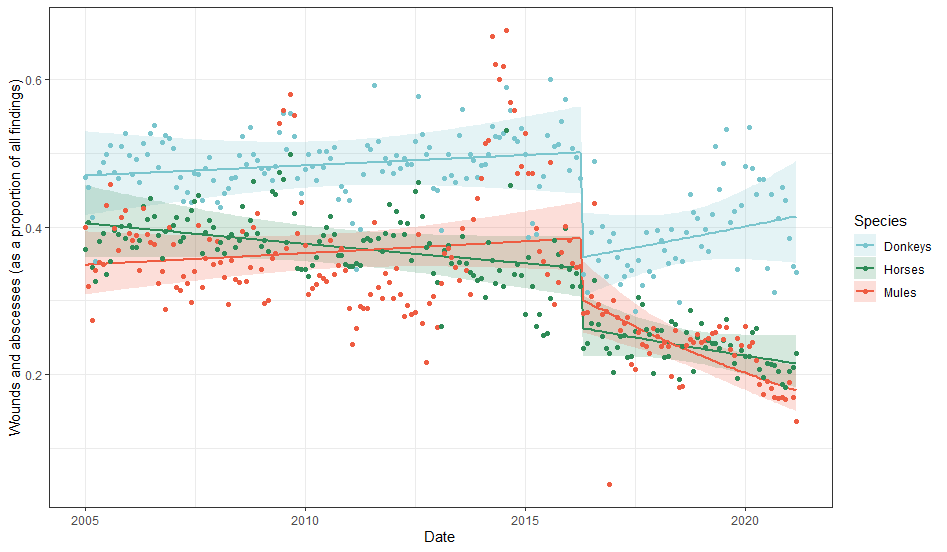


Supplement 5 Figure 1 - Negative binomial regression model illustrating the proportion of wounds and abscesses in relation to all clinical finding categories by species over time, adjusted for time and changes in reporting template, in a retrospective study of clinical findings of working equids in low- and middle-income countries presenting to an international NGO between January 2005 and March 2021


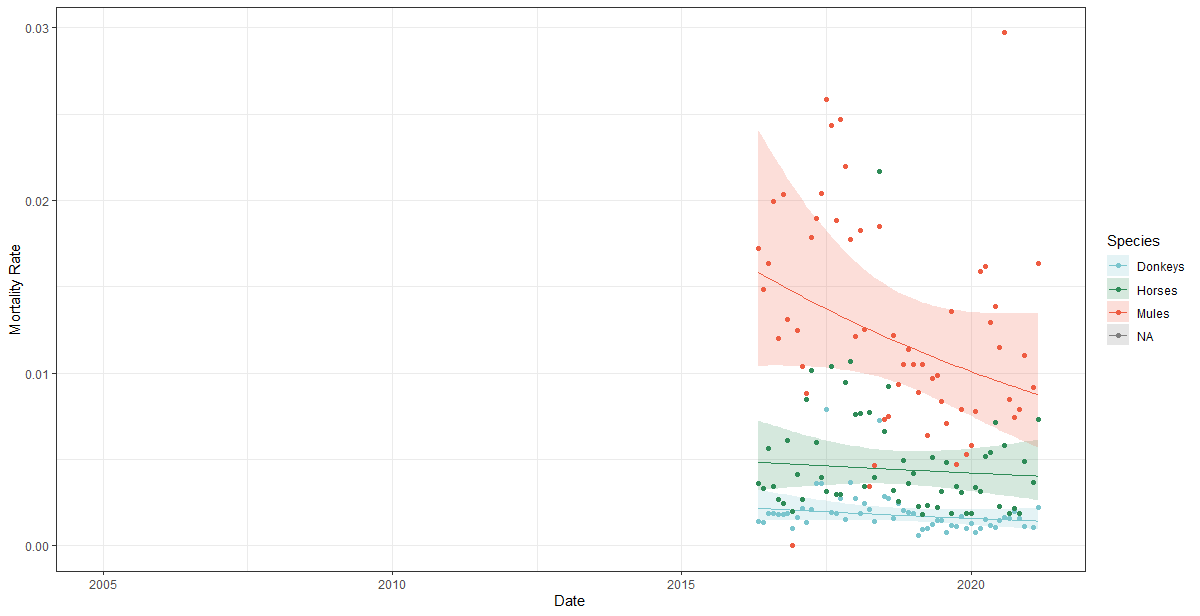


Supplement 5 Figure 2 - Negative binomial regression model illustrating the mortality risk per equid species over time in a retrospective study of clinical findings of working equids in low- and middle-income countries presenting to an international NGO. Data available from May 2016 to March 2021 only. An outlier has been removed for visualisation purposes.
